# 2-Eth­oxy-6-({2-[(3-eth­oxy-2-hy­droxy­benzyl­idene)amino]­benz­yl}imino­meth­yl)phenol

**DOI:** 10.1107/S1600536812031479

**Published:** 2012-07-18

**Authors:** K. U. Ambili, S. S. Sreejith, Jinsa Mary Jacob, M. Sithambaresan, M. R. Prathapachandra Kurup

**Affiliations:** a Department of Applied Chemistry, Cochin University of Science and Technology, Kochi 682 022, India; b Department of Chemistry, Faculty of Science, Eastern University, Sri Lanka, Chenkalady, Sri Lanka

## Abstract

The title compound, C_25_H_26_N_2_O_4_, exists in an *E* conformation with respect to each azomethine link. The two phenol-substituted benzene rings are twisted away from the plane of the diimine benzene ring by dihedral angles of 27.25 (5) and 56.67 (5)°. The mol­ecular structure is stabilized by intra­molecular O—H⋯N hydrogen bonds.

## Related literature

For the applications of salen Schiff bases, see: Cozzi (2004 ▶); Hodnett & Dunn (1970 ▶). For the synthesis of Schiff bases, see: Tümer (2000 ▶). For a related structure, see: Aslantaş *et al.* (2007 ▶).
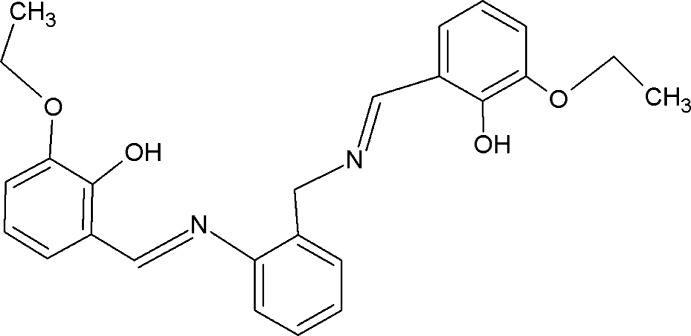


## Experimental

### 

#### Crystal data

C_25_H_26_N_2_O_4_
*M* *_r_* = 418.48
Monoclinic, 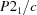

*a* = 4.8315 (4) Å
*b* = 17.5414 (14) Å
*c* = 25.828 (2) Å
β = 94.356 (3)°
*V* = 2182.6 (3) Å^3^
*Z* = 4
Mo *K*α radiation
μ = 0.09 mm^−1^
*T* = 296 K
0.40 × 0.20 × 0.20 mm

#### Data collection

Bruker Kappa APEXII CCD diffractometer
Absorption correction: multi-scan (*SADABS*; Bruker, 2004 ▶) *T*_min_ = 0.979, *T*_max_ = 0.983
32150 measured reflections
3833 independent reflections
2543 reflections with *I* > 2σ(*I*)
*R*_int_ = 0.044

#### Refinement

*R*[*F* ^2^ > 2σ(*F* ^2^)] = 0.038
*wR*(*F* ^2^) = 0.112
*S* = 1.05
3833 reflections
291 parameters
2 restraints
H atoms treated by a mixture of independent and constrained refinement
Δρ_max_ = 0.13 e Å^−3^
Δρ_min_ = −0.13 e Å^−3^

### 

Data collection: *APEX2* (Bruker, 2004 ▶); cell refinement: *APEX2* and *SAINT* (Bruker, 2004 ▶); data reduction: *SAINT* and *XPREP* (Bruker, 2004 ▶); program(s) used to solve structure: *SHELXS97* (Sheldrick, 2008 ▶); program(s) used to refine structure: *SHELXL97* (Sheldrick, 2008 ▶); molecular graphics: *ORTEP-3* (Farrugia, 1997 ▶); software used to prepare material for publication: *SHELXL97* and *publCIF* (Westrip, 2010 ▶).

## Supplementary Material

Crystal structure: contains datablock(s) global, I. DOI: 10.1107/S1600536812031479/fj2580sup1.cif

Structure factors: contains datablock(s) I. DOI: 10.1107/S1600536812031479/fj2580Isup2.hkl

Supplementary material file. DOI: 10.1107/S1600536812031479/fj2580Isup3.cml

Additional supplementary materials:  crystallographic information; 3D view; checkCIF report

## Figures and Tables

**Table 1:** Hydrogen-bond geometry (Å, °)

*D*—H⋯*A*	*D*—H	H⋯*A*	*D*⋯*A*	*D*—H⋯*A*
O2—H2O⋯N1	0.85 (1)	1.79 (1)	2.5711 (19)	151 (2)
O3—H3O⋯N2	0.85 (1)	1.80 (1)	2.5844 (19)	152 (2)

**Fig. 1.**
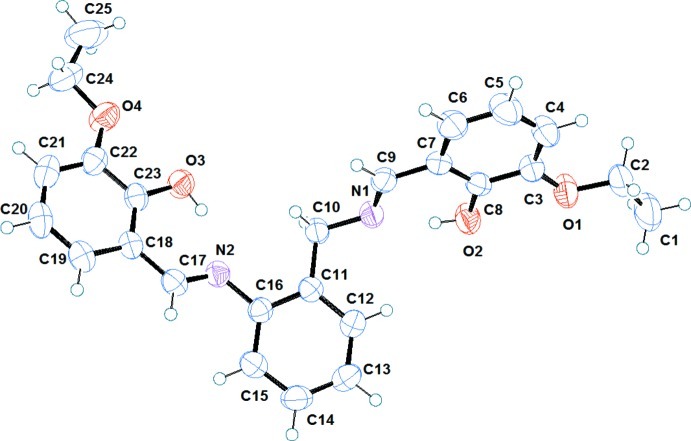
ORTEP view of the unique part of the compound, drawn with 50% probability displacement ellipsoids for the non-H atoms.

**Fig. 2.**
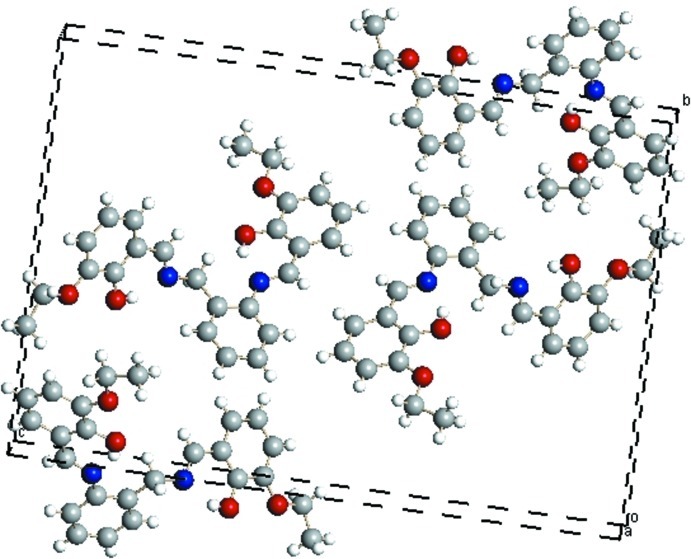
Packing diagram of the compound viewed along a axis.

## supplementary crystallographic information

### Comment

The chelating structure, moderate electron donation and easy tunable electronic and steric effects make dicompartmental salen type Schiff bases to act as versatile ligands. They are able to stabilize different metals in various oxidation states and control the performance of the metals in various catalytic transformations (Cozzi, 2004). Schiff bases are very selective in the sense that they provide geometrical cavity control for host guest interaction and modulate its lipophilicity to stabilize a specific metal ion. Moreover, it has been suggested that the azomethine linkage in Schiff bases is responsible for their biological activities such as antimicrobial, antifungal and antitumor and also to be used as herbicides (Hodnett & Dunn, 1970).

The *E* conformation of the compound is evidenced from the torsion angles, 176.94 (14)° and 179.38 (15)° made by the C10—N1—C9—C7 and C16—N2—C17—C18 linkages respectively. The bond lengths and bond angles are in normal ranges and agree with the related structure (Aslantaş, *et al.*, 2007). The crystal involves two intramolecular O—H···N hydrogen bonds and C—H···π interactions which make the molecule stable.

### Experimental

The title compound was prepared according to the reported procedure (Tümer, 2000) by the condensation of the ethanolic solution of 3-ethoxy-2-hydroxybenzaldehyde (1 mmol, 0.166 g) with an ethanolic solution of 2-aminobenzylamine (0.5 mmol, 0.061 g). The reaction mixture was heated to reflux for 6 h and kept for cooling at room temperature. The slow evaporation yielded orange-yellow crystals of *N,N'*-bis(3-ethoxy-2-hydroxybenzylidene)-2-aminobenzylamine.

### Refinement

All H atoms on C were placed in calculated positions, guided by difference maps, with C—H bond distances 0.93–0.97 Å. H atoms were assigned as *U*_iso_=1.2Ueq (1.5 for Me). O(2)—H(2o) and O(3)—H(3o) H atoms were located from difference maps and restrained using *DFIX* instructions.

### Crystal data

**Table:**

C_25_H_26_N_2_O_4_	*F*(000) = 888
*M**_r_* = 418.48	*D*_x_ = 1.273 Mg m^−^^3^
Monoclinic, *P*2_1_/*c*	Mo *K*α radiation, λ = 0.71073 Å
Hall symbol: -P 2ybc	Cell parameters from 5446 reflections
*a* = 4.8315 (4) Å	θ = 2.3–22.4°
*b* = 17.5414 (14) Å	µ = 0.09 mm^−^^1^
*c* = 25.828 (2) Å	*T* = 296 K
β = 94.356 (3)°	Needle-like, orange
*V* = 2182.6 (3) Å^3^	0.40 × 0.20 × 0.20 mm
*Z* = 4	

### Data collection

**Table:**

Bruker Kappa APEXII CCD diffractometer	3833 independent reflections
Radiation source: fine-focus sealed tube	2543 reflections with *I* > 2σ(*I*)
Graphite monochromator	*R*_int_ = 0.044
ω and φ scan	θ_max_ = 25.0°, θ_min_ = 2.5°
Absorption correction: multi-scan (*SADABS*; Bruker, 2004)	*h* = −5→5
*T*_min_ = 0.979, *T*_max_ = 0.983	*k* = −20→20
32150 measured reflections	*l* = −30→30

### Refinement

**Table:**

Refinement on *F*^2^	Secondary atom site location: difference Fourier map
Least-squares matrix: full	Hydrogen site location: inferred from neighbouring sites
*R*[*F*^2^ > 2σ(*F*^2^)] = 0.038	H atoms treated by a mixture of independent and constrained refinement
*wR*(*F*^2^) = 0.112	*w* = 1/[σ^2^(*F*_o_^2^) + (0.0493*P*)^2^ + 0.3451*P*] where *P* = (*F*_o_^2^ + 2*F*_c_^2^)/3
*S* = 1.05	(Δ/σ)_max_ = 0.001
3833 reflections	Δρ_max_ = 0.13 e Å^−^^3^
291 parameters	Δρ_min_ = −0.13 e Å^−^^3^
2 restraints	Extinction correction: *SHELXL97* (Sheldrick, 2008), Fc^*^=kFc[1+0.001xFc^2^λ^3^/sin(2θ)]^-1/4^
Primary atom site location: structure-invariant direct methods	Extinction coefficient: 0.0062 (9)

### Special details

**Table:**

Geometry. All e.s.d.'s (except the e.s.d. in the dihedral angle between two l.s. planes) are estimated using the full covariance matrix. The cell e.s.d.'s are taken into account individually in the estimation of e.s.d.'s in distances, angles and torsion angles; correlations between e.s.d.'s in cell parameters are only used when they are defined by crystal symmetry. An approximate (isotropic) treatment of cell e.s.d.'s is used for estimating e.s.d.'s involving l.s. planes.
Refinement. Refinement of *F*^2^ against ALL reflections. The weighted *R*-factor *wR* and goodness of fit *S* are based on *F*^2^, conventional *R*-factors *R* are based on *F*, with *F* set to zero for negative *F*^2^. The threshold expression of *F*^2^ > σ(*F*^2^) is used only for calculating *R*-factors(gt) *etc*. and is not relevant to the choice of reflections for refinement. *R*-factors based on *F*^2^ are statistically about twice as large as those based on *F*, and *R*- factors based on ALL data will be even larger.

### Fractional atomic coordinates and isotropic or equivalent isotropic displacement parameters (Å^2^)

**Table:**

	*x*	*y*	*z*	*U*_iso_*/*U*_eq_	
O1	1.5523 (3)	1.08404 (8)	0.43051 (5)	0.0645 (4)	
O2	1.1570 (3)	1.09379 (8)	0.35604 (5)	0.0598 (4)	
O3	0.0532 (3)	0.93676 (8)	0.17006 (5)	0.0543 (4)	
O4	−0.3152 (3)	0.82995 (8)	0.15297 (5)	0.0659 (4)	
N1	0.8660 (3)	1.03221 (9)	0.27970 (5)	0.0466 (4)	
N2	0.4050 (3)	1.03122 (8)	0.13518 (5)	0.0445 (4)	
C1	1.8136 (6)	1.16138 (15)	0.48999 (10)	0.0968 (9)	
H1A	1.8577	1.1960	0.4630	0.145*	
H1B	1.9602	1.1620	0.5172	0.145*	
H1C	1.6429	1.1768	0.5037	0.145*	
C2	1.7824 (4)	1.08294 (13)	0.46832 (8)	0.0634 (6)	
H2A	1.9497	1.0679	0.4524	0.076*	
H2B	1.7496	1.0469	0.4957	0.076*	
C3	1.4950 (4)	1.01888 (11)	0.40288 (7)	0.0479 (5)	
C4	1.6259 (4)	0.94994 (12)	0.41106 (8)	0.0589 (5)	
H4	1.7682	0.9453	0.4372	0.071*	
C5	1.5485 (5)	0.88751 (12)	0.38081 (8)	0.0669 (6)	
H5	1.6380	0.8411	0.3869	0.080*	
C6	1.3413 (4)	0.89335 (12)	0.34193 (8)	0.0582 (5)	
H6	1.2913	0.8510	0.3217	0.070*	
C7	1.2044 (3)	0.96269 (10)	0.33256 (6)	0.0433 (4)	
C8	1.2814 (3)	1.02555 (10)	0.36311 (7)	0.0431 (4)	
C9	0.9881 (4)	0.96941 (11)	0.29046 (7)	0.0464 (5)	
H9	0.9376	0.9265	0.2708	0.056*	
C10	0.6616 (3)	1.03497 (11)	0.23529 (6)	0.0471 (5)	
H10A	0.4828	1.0494	0.2470	0.057*	
H10B	0.6430	0.9847	0.2198	0.057*	
C11	0.7426 (3)	1.09114 (10)	0.19477 (7)	0.0413 (4)	
C12	0.9465 (4)	1.14566 (11)	0.20497 (7)	0.0503 (5)	
H12	1.0399	1.1475	0.2378	0.060*	
C13	1.0149 (4)	1.19732 (11)	0.16787 (8)	0.0583 (5)	
H13	1.1529	1.2334	0.1756	0.070*	
C14	0.8783 (4)	1.19519 (11)	0.11950 (8)	0.0610 (6)	
H14	0.9212	1.2305	0.0945	0.073*	
C15	0.6775 (4)	1.14086 (11)	0.10774 (7)	0.0550 (5)	
H15	0.5866	1.1394	0.0747	0.066*	
C16	0.6101 (3)	1.08823 (10)	0.14499 (7)	0.0419 (4)	
C17	0.3525 (4)	1.00201 (11)	0.09022 (7)	0.0468 (5)	
H17	0.4510	1.0191	0.0629	0.056*	
C18	0.1456 (3)	0.94344 (10)	0.08038 (7)	0.0438 (4)	
C19	0.0901 (4)	0.91471 (12)	0.03008 (7)	0.0565 (5)	
H19	0.1824	0.9347	0.0028	0.068*	
C20	−0.0987 (4)	0.85754 (13)	0.02086 (8)	0.0636 (6)	
H20	−0.1334	0.8385	−0.0126	0.076*	
C21	−0.2384 (4)	0.82791 (12)	0.06090 (9)	0.0604 (6)	
H21	−0.3671	0.7891	0.0541	0.072*	
C22	−0.1905 (4)	0.85472 (11)	0.11067 (7)	0.0493 (5)	
C23	0.0053 (3)	0.91322 (10)	0.12070 (7)	0.0430 (4)	
C24	−0.4979 (4)	0.76620 (12)	0.14644 (10)	0.0709 (6)	
H24A	−0.3996	0.7224	0.1341	0.085*	
H24B	−0.6513	0.7780	0.1213	0.085*	
C25	−0.6032 (6)	0.74931 (17)	0.19800 (12)	0.1079 (10)	
H25A	−0.4492	0.7407	0.2230	0.162*	
H25B	−0.7181	0.7046	0.1954	0.162*	
H25C	−0.7102	0.7918	0.2088	0.162*	
H2O	1.031 (4)	1.0877 (14)	0.3316 (6)	0.090 (8)*	
H3O	0.172 (4)	0.9724 (10)	0.1691 (10)	0.103 (9)*	

### Atomic displacement parameters (Å^2^)

**Table:**

	*U*^11^	*U*^22^	*U*^33^	*U*^12^	*U*^13^	*U*^23^
O1	0.0646 (9)	0.0605 (9)	0.0640 (9)	0.0055 (7)	−0.0227 (7)	−0.0080 (7)
O2	0.0650 (9)	0.0476 (9)	0.0632 (9)	0.0063 (7)	−0.0189 (7)	−0.0030 (7)
O3	0.0597 (8)	0.0607 (9)	0.0431 (8)	−0.0100 (7)	0.0075 (6)	−0.0064 (7)
O4	0.0606 (8)	0.0640 (10)	0.0739 (10)	−0.0166 (7)	0.0109 (7)	−0.0022 (8)
N1	0.0454 (8)	0.0537 (10)	0.0400 (8)	−0.0027 (7)	−0.0006 (7)	0.0008 (7)
N2	0.0416 (8)	0.0480 (9)	0.0432 (9)	0.0004 (7)	−0.0005 (6)	0.0010 (7)
C1	0.115 (2)	0.0734 (18)	0.0933 (19)	−0.0083 (15)	−0.0501 (16)	−0.0043 (15)
C2	0.0626 (12)	0.0727 (16)	0.0519 (12)	−0.0012 (11)	−0.0152 (10)	0.0006 (11)
C3	0.0471 (10)	0.0514 (13)	0.0448 (11)	−0.0009 (9)	−0.0006 (8)	0.0009 (9)
C4	0.0594 (12)	0.0619 (14)	0.0536 (12)	0.0102 (10)	−0.0080 (10)	0.0044 (11)
C5	0.0766 (14)	0.0535 (14)	0.0691 (14)	0.0177 (11)	−0.0043 (12)	0.0057 (12)
C6	0.0705 (13)	0.0456 (12)	0.0576 (13)	0.0023 (10)	0.0004 (10)	−0.0035 (10)
C7	0.0465 (10)	0.0436 (11)	0.0400 (10)	−0.0036 (8)	0.0043 (8)	0.0056 (9)
C8	0.0451 (10)	0.0412 (11)	0.0428 (10)	0.0020 (8)	0.0031 (8)	0.0063 (9)
C9	0.0510 (10)	0.0495 (12)	0.0391 (10)	−0.0096 (9)	0.0060 (8)	−0.0006 (9)
C10	0.0421 (9)	0.0580 (12)	0.0405 (10)	−0.0053 (8)	−0.0009 (8)	0.0031 (9)
C11	0.0383 (9)	0.0430 (10)	0.0425 (10)	0.0024 (8)	0.0036 (7)	0.0001 (8)
C12	0.0482 (10)	0.0503 (12)	0.0517 (11)	−0.0047 (9)	0.0001 (9)	−0.0025 (10)
C13	0.0593 (12)	0.0485 (12)	0.0672 (14)	−0.0123 (10)	0.0059 (10)	−0.0003 (10)
C14	0.0736 (14)	0.0491 (13)	0.0609 (14)	−0.0082 (10)	0.0093 (11)	0.0105 (10)
C15	0.0641 (12)	0.0540 (13)	0.0463 (11)	−0.0020 (10)	−0.0005 (9)	0.0061 (10)
C16	0.0388 (9)	0.0429 (11)	0.0440 (10)	0.0007 (8)	0.0030 (8)	0.0002 (8)
C17	0.0482 (10)	0.0528 (12)	0.0394 (11)	0.0020 (9)	0.0032 (8)	0.0031 (9)
C18	0.0439 (9)	0.0472 (11)	0.0394 (10)	0.0044 (8)	−0.0025 (8)	−0.0033 (9)
C19	0.0628 (12)	0.0649 (14)	0.0411 (11)	0.0010 (11)	−0.0010 (9)	−0.0038 (10)
C20	0.0668 (13)	0.0709 (15)	0.0508 (13)	0.0024 (12)	−0.0115 (10)	−0.0182 (11)
C21	0.0501 (11)	0.0577 (13)	0.0712 (15)	−0.0023 (10)	−0.0094 (10)	−0.0147 (12)
C22	0.0408 (9)	0.0494 (12)	0.0573 (13)	0.0023 (9)	0.0011 (9)	−0.0023 (10)
C23	0.0402 (9)	0.0468 (11)	0.0414 (11)	0.0059 (8)	−0.0009 (8)	−0.0045 (9)
C24	0.0572 (12)	0.0480 (13)	0.1072 (19)	−0.0059 (10)	0.0038 (12)	0.0088 (12)
C25	0.099 (2)	0.093 (2)	0.134 (2)	−0.0265 (16)	0.0211 (18)	0.0369 (18)

### Geometric parameters (Å, º)

**Table:**

O1—C3	1.365 (2)	C10—H10A	0.9700
O1—C2	1.423 (2)	C10—H10B	0.9700
O2—C8	1.346 (2)	C11—C12	1.384 (2)
O2—H2O	0.8502 (11)	C11—C16	1.393 (2)
O3—C23	1.343 (2)	C12—C13	1.377 (3)
O3—H3O	0.8501 (11)	C12—H12	0.9300
O4—C22	1.358 (2)	C13—C14	1.368 (3)
O4—C24	1.427 (2)	C13—H13	0.9300
N1—C9	1.270 (2)	C14—C15	1.377 (3)
N1—C10	1.456 (2)	C14—H14	0.9300
N2—C17	1.277 (2)	C15—C16	1.390 (2)
N2—C16	1.417 (2)	C15—H15	0.9300
C1—C2	1.489 (3)	C17—C18	1.443 (2)
C1—H1A	0.9600	C17—H17	0.9300
C1—H1B	0.9600	C18—C23	1.390 (2)
C1—H1C	0.9600	C18—C19	1.400 (2)
C2—H2A	0.9700	C19—C20	1.364 (3)
C2—H2B	0.9700	C19—H19	0.9300
C3—C4	1.374 (3)	C20—C21	1.379 (3)
C3—C8	1.404 (2)	C20—H20	0.9300
C4—C5	1.380 (3)	C21—C22	1.372 (3)
C4—H4	0.9300	C21—H21	0.9300
C5—C6	1.367 (3)	C22—C23	1.407 (2)
C5—H5	0.9300	C24—C25	1.491 (4)
C6—C7	1.397 (3)	C24—H24A	0.9700
C6—H6	0.9300	C24—H24B	0.9700
C7—C8	1.390 (2)	C25—H25A	0.9600
C7—C9	1.455 (2)	C25—H25B	0.9600
C9—H9	0.9300	C25—H25C	0.9600
C10—C11	1.510 (2)		
			
C3—O1—C2	117.69 (15)	C13—C12—C11	121.82 (18)
C8—O2—H2O	106.0 (17)	C13—C12—H12	119.1
C23—O3—H3O	105.4 (18)	C11—C12—H12	119.1
C22—O4—C24	117.63 (16)	C14—C13—C12	119.52 (18)
C9—N1—C10	118.48 (16)	C14—C13—H13	120.2
C17—N2—C16	122.26 (15)	C12—C13—H13	120.2
C2—C1—H1A	109.5	C13—C14—C15	120.20 (19)
C2—C1—H1B	109.5	C13—C14—H14	119.9
H1A—C1—H1B	109.5	C15—C14—H14	119.9
C2—C1—H1C	109.5	C14—C15—C16	120.34 (18)
H1A—C1—H1C	109.5	C14—C15—H15	119.8
H1B—C1—H1C	109.5	C16—C15—H15	119.8
O1—C2—C1	107.27 (18)	C15—C16—C11	119.93 (16)
O1—C2—H2A	110.3	C15—C16—N2	122.85 (16)
C1—C2—H2A	110.3	C11—C16—N2	117.21 (15)
O1—C2—H2B	110.3	N2—C17—C18	122.22 (16)
C1—C2—H2B	110.3	N2—C17—H17	118.9
H2A—C2—H2B	108.5	C18—C17—H17	118.9
O1—C3—C4	125.73 (17)	C23—C18—C19	119.22 (17)
O1—C3—C8	114.93 (16)	C23—C18—C17	120.68 (16)
C4—C3—C8	119.35 (18)	C19—C18—C17	120.07 (17)
C3—C4—C5	120.62 (18)	C20—C19—C18	120.32 (19)
C3—C4—H4	119.7	C20—C19—H19	119.8
C5—C4—H4	119.7	C18—C19—H19	119.8
C6—C5—C4	120.56 (19)	C19—C20—C21	120.34 (19)
C6—C5—H5	119.7	C19—C20—H20	119.8
C4—C5—H5	119.7	C21—C20—H20	119.8
C5—C6—C7	120.20 (19)	C22—C21—C20	121.08 (19)
C5—C6—H6	119.9	C22—C21—H21	119.5
C7—C6—H6	119.9	C20—C21—H21	119.5
C8—C7—C6	119.32 (16)	O4—C22—C21	126.19 (18)
C8—C7—C9	120.42 (16)	O4—C22—C23	114.74 (16)
C6—C7—C9	120.25 (17)	C21—C22—C23	119.07 (18)
O2—C8—C7	122.08 (15)	O3—C23—C18	122.37 (16)
O2—C8—C3	117.98 (16)	O3—C23—C22	117.65 (16)
C7—C8—C3	119.95 (17)	C18—C23—C22	119.97 (16)
N1—C9—C7	121.94 (17)	O4—C24—C25	107.5 (2)
N1—C9—H9	119.0	O4—C24—H24A	110.2
C7—C9—H9	119.0	C25—C24—H24A	110.2
N1—C10—C11	111.81 (14)	O4—C24—H24B	110.2
N1—C10—H10A	109.3	C25—C24—H24B	110.2
C11—C10—H10A	109.3	H24A—C24—H24B	108.5
N1—C10—H10B	109.3	C24—C25—H25A	109.5
C11—C10—H10B	109.3	C24—C25—H25B	109.5
H10A—C10—H10B	107.9	H25A—C25—H25B	109.5
C12—C11—C16	118.15 (16)	C24—C25—H25C	109.5
C12—C11—C10	122.49 (16)	H25A—C25—H25C	109.5
C16—C11—C10	119.36 (15)	H25B—C25—H25C	109.5
			
C3—O1—C2—C1	175.91 (19)	C14—C15—C16—C11	1.1 (3)
C2—O1—C3—C4	5.6 (3)	C14—C15—C16—N2	179.84 (17)
C2—O1—C3—C8	−174.61 (16)	C12—C11—C16—C15	−2.1 (2)
O1—C3—C4—C5	179.47 (19)	C10—C11—C16—C15	178.31 (17)
C8—C3—C4—C5	−0.4 (3)	C12—C11—C16—N2	179.06 (15)
C3—C4—C5—C6	0.5 (3)	C10—C11—C16—N2	−0.5 (2)
C4—C5—C6—C7	−0.3 (3)	C17—N2—C16—C15	30.6 (3)
C5—C6—C7—C8	0.1 (3)	C17—N2—C16—C11	−150.65 (17)
C5—C6—C7—C9	178.64 (18)	C16—N2—C17—C18	179.38 (15)
C6—C7—C8—O2	−179.86 (16)	N2—C17—C18—C23	−3.8 (3)
C9—C7—C8—O2	1.6 (3)	N2—C17—C18—C19	178.20 (17)
C6—C7—C8—C3	0.0 (3)	C23—C18—C19—C20	−0.2 (3)
C9—C7—C8—C3	−178.53 (15)	C17—C18—C19—C20	177.81 (17)
O1—C3—C8—O2	0.2 (2)	C18—C19—C20—C21	0.5 (3)
C4—C3—C8—O2	179.99 (17)	C19—C20—C21—C22	−0.3 (3)
O1—C3—C8—C7	−179.72 (15)	C24—O4—C22—C21	5.1 (3)
C4—C3—C8—C7	0.1 (3)	C24—O4—C22—C23	−174.61 (16)
C10—N1—C9—C7	176.94 (14)	C20—C21—C22—O4	−179.98 (19)
C8—C7—C9—N1	1.0 (3)	C20—C21—C22—C23	−0.2 (3)
C6—C7—C9—N1	−177.52 (17)	C19—C18—C23—O3	178.33 (16)
C9—N1—C10—C11	−119.33 (18)	C17—C18—C23—O3	0.3 (3)
N1—C10—C11—C12	−14.9 (2)	C19—C18—C23—C22	−0.3 (3)
N1—C10—C11—C16	164.62 (15)	C17—C18—C23—C22	−178.31 (16)
C16—C11—C12—C13	1.6 (3)	O4—C22—C23—O3	1.6 (2)
C10—C11—C12—C13	−178.91 (17)	C21—C22—C23—O3	−178.17 (16)
C11—C12—C13—C14	0.1 (3)	O4—C22—C23—C18	−179.72 (16)
C12—C13—C14—C15	−1.2 (3)	C21—C22—C23—C18	0.5 (3)
C13—C14—C15—C16	0.6 (3)	C22—O4—C24—C25	178.99 (18)

### Hydrogen-bond geometry (Å, º)

**Table:**

*D*—H···*A*	*D*—H	H···*A*	*D*···*A*	*D*—H···*A*
O2—H2*O*···N1	0.85 (1)	1.79 (1)	2.5711 (19)	151 (2)
O3—H3*O*···N2	0.85 (1)	1.80 (1)	2.5844 (19)	152 (2)
